# pTC Plasmids from *Sulfolobus* Species in the Geothermal Area of Tengchong, China: Genomic Conservation and Naturally-Occurring Variations as a Result of Transposition by Mobile Genetic Elements

**DOI:** 10.3390/life5010506

**Published:** 2015-02-12

**Authors:** Xiaoyu Xiang, Xiaoxing Huang, Haina Wang, Li Huang

**Affiliations:** State Key Laboratory of Microbial Resources, Institute of Microbiology, Chinese Academy of Sciences, No. 1 West Beichen Road, Chaoyang District, Beijing 100101, China; E-Mails: xiangxiaoyu@yahoo.com (X.X.); xiaoxinghuang@yeah.net (X.H.); wanghaine@163.com (H.W.)

**Keywords:** pTC plasmids, conjugative plasmids, genome sequence, mobile genetic elements, *Sulfolobus tengchongensis*

## Abstract

Plasmids occur frequently in Archaea. A novel plasmid (denoted pTC1) containing typical conjugation functions has been isolated from *Sulfolobus tengchongensis* RT8-4, a strain obtained from a hot spring in Tengchong, China, and characterized. The plasmid is a circular double-stranded DNA molecule of 20,417 bp. Among a total of 26 predicted pTC1 ORFs, 23 have homologues in other known *Sulfolobus* conjugative plasmids (CPs). pTC1 resembles other *Sulfolobus* CPs in genome architecture, and is most highly conserved in the genomic region encoding conjugation functions. However, attempts to demonstrate experimentally the capacity of the plasmid for conjugational transfer were unsuccessful. A survey revealed that pTC1 and its closely related plasmid variants were widespread in the geothermal area of Tengchong. Variations of the plasmids at the target sites for transposition by an insertion sequence (IS) and a miniature inverted-repeat transposable element (MITE) were readily detected. The IS was efficiently inserted into the pTC1 genome, and the inserted sequence was inactivated and degraded more frequently in an imprecise manner than in a precise manner. These results suggest that the host organism has evolved a strategy to maintain a balance between the insertion and elimination of mobile genetic elements to permit genomic plasticity while inhibiting their fast spreading.

## 1. Introduction

Plasmids occur widely in archaea. Research on archaeal plasmids has been focused on a narrow range of archaea including members of Sulfolobaceae, Haloarchaeaceae, Thermococcaceae and various methanogens. So far, over 60 plasmids have been isolated from archaea. Among them, a total of 24 plasmids or plasmid virus hybrids are derived from Sulfolobaceae, with all but two (pDL10 and pAH1 from *Acidianus*) from *Sulfolobus* species, and, therefore, they are known as *Sulfolobus* plasmids. *Sulfolobus* plasmids include primarily cryptic and conjugative plasmids (CPs). All known archaeal CPs (13 in total) were isolated from species of Sulfolobaceae. The first *Sulfolobus* CP, pNOB8, was isolated from *Sulfolobus* sp. NOB8H2, a strain obtained from a hotspring in Japan [1,2]. Later, pING family plasmids (pKEF9, pHVE14, pARN3 and pARN4) and pSOG family plasmids were isolated from different *S. islandicus* isolates derived from Icelandic hotspring samples [3,4,5]. Recently, pAH1 was obtained from *Acidianus hospitalis* [6]. A survey shows that up to 3% of the *Sulfolobus* isolates from terrestrial hotsprings in Iceland contain a CP [5].

Comparative genomic analysis reveals the architectural conservation among *Sulfolobus* CPs of different geographical origins. The genomes of the CPs are characterized by the presence of three functionally distinct regions, *i.e.*, sections A, B and C [5,7]. Section A encodes conjugative functions. Proteins encoded by section A ORFs are transmembrane proteins presumably involved in DNA transfer. They include highly conserved homologues of the bacterial conjugative proteins TraG and TrbE [2]. Section B contains a putative origin of replication with characteristic sequence repeats. Section C is proposed to encode proteins for the initiation and regulation of plasmid replication [7]. Despite the availability of increasing genomic data on *Sulfolobus* CPs, little is known about the molecular mechanisms of replication and host interactions of these plasmids.

Known *Sulfolobus* CPs have shown striking variation in genome stability. While some of the *Sulfolobus* CPs (e.g., pAH1, pNOB8, pSOG2) are relatively stable, at least in their natural host, others (e.g., pNOB8-33, pING1) are extremely unstable. A number of mechanisms, including the host defense systems such as the CRISPR-CAS systems, homologous recombination and transposition by mobile genetic elements (e.g., ISs), have been proposed to be responsible for the instability of the CPs [2,7,8,9]. In any case, the plasticity of *Sulfolobus* CPs likely results from CP–host interactions which contribute to the co-evolution of plasmids and their archaeal hosts.

Here, we report the isolation and characterization of the conjugative plasmid pTC1 from *Sulfolobus tengchongensis* RT8-4, a strain obtained from the hot spring in Tengchong, Yunnan, China. The genome sequence of pTC1 was determined and compared with other known *Sulfolobus* CPs. We also conducted a survey on pTC1 and its plasmid variants in the geothermal area of Tengchong. We found that pTC plasmids were widespread in the area of survey. Genomic variations of the pTC plasmids as a result of transposition by an IS or an MITE were characterized to gain insight into the generation of the diversity of the plasmids.

## 2. Materials and Methods

### 2.1. Sample Collection and Screening for Plasmids

Water and sediment samples were collected from hot springs and mud holes with temperatures ranging from 61 to 94 °C and pH from 2.0 to 6.0 in a geothermal area in Tengchong, Yunnan, China, and transported in sterile 50-mL centrifuge tubes at ambient temperature. An aliquot of each sample (1–2 mL of spring water or 1–2 g of mud) was inoculated into Zillig’s medium (100 mL) supplemented with 0.2% tryptone [3]. Incubation was carried out at 80 °C with moderate shaking. When microbial growth became apparent (1–2 weeks), the enrichment culture was tested for the presence of covalently closed circular DNA (cccDNA) by using the alkaline lysis method [10]. If cccDNA was detected, the enrichment culture was streaked onto 0.8% Gelrite plates containing Zillig’s medium supplemented with 0.2% tryptone. The plates were incubated for 5–7 days at 80 °C. Single colonies formed on the plates were picked and grown in Zillig’s liquid medium. The presence of plasmids was determined by extraction of the total DNA by alkaline lysis, restriction digestion of the DNA and agarose gel electrophoresis of the restriction digests [3,4,11]. Isolates containing plasmids were identified by amplifying the 16S rRNA gene with a pair of Archaea-specific primers [12] and subsequent sequencing. The culture was also subjected to electron microscopy to determine if the extrachromosomal genetic element was a plasmid or a virus. *S. tengchongensis* strain RT8-4 [13] was also tested as described above for the presence of plasmids.

### 2.2. Electron Microscopy

Samples were applied to carbon grids, negatively stained with 2% uranyl acetate and examined under a HitachiH-600A electron microscope [13].

### 2.3. DNA Sequencing and Genome Analysis

Plasmid pTC1 DNA was extracted from *S. tengchongensis* RT8-4 by the alkaline lysis method and purified using a plasmid purification kit (Biodev Co., Beijing, China). The purified plasmid DNA was digested with *Eco*RI. Restriction fragments were purified by electrophoresis in 1% agarose and subsequent extraction using a Gel Extraction Kit (QIAGEN), and cloned into pBluescript KSII. Clones containing plasmid fragments were collected, and the inserts were sequenced from both ends. PCR reactions were conducted to confirm the linking relationships of the fragments and to fill gaps. Sequence assembly was performed using DNASTAR. Open reading frames (ORFs) encoding polypeptides of >60 amino acid residues in size were identified by the ORF Finder at NCBI. Two ORFs that code for polypeptides shorter than 60 amino acid residues were also identified because of their high E-values. ORFs were searched against the GenBank by BLASTP at NCBI. The sequence of pTC1 has been deposited in GenBank (Accession Number NC_005969.1).

### 2.4. Detection of Transposition by ISSte1 and MITE at the Specific Sites

Enrichment cultures were established with hot spring and mud hole samples as described above. Cells were harvested from each enrichment culture by centrifugation. Total DNA was isolated by phenol/chloroform extraction. Plasmid DNA was extracted by using the alkaline lysis method. PCR amplification was carried out on the total DNA or the plasmid DNA using either the primer pair 5'-TTGGATTATCATTAGCATTTTGTCATC/5'-CTTCAATAACCTCAATAAGCCCC for the *ISSte1* site or the primer pair 5'-CACCCCATTGAGTGGACGAT/5'-CCTCATCTACTCTTTACAG for the MITE site. Products shorter than expected for the insertion of the full-length *ISSte1* or MITE were cloned into pEasy-T1 (TransGen Biotech, Beijing, China). Following transformation of the resulting plasmids into *E. coli* DH5α (TransGen Biotech), transformants were selected and grown in liquid LB medium. The plasmids were isolated, and the inserts were sequenced.

## 3. Results

### 3.1. Identification of Plasmid pTC1

**Figure 1:**
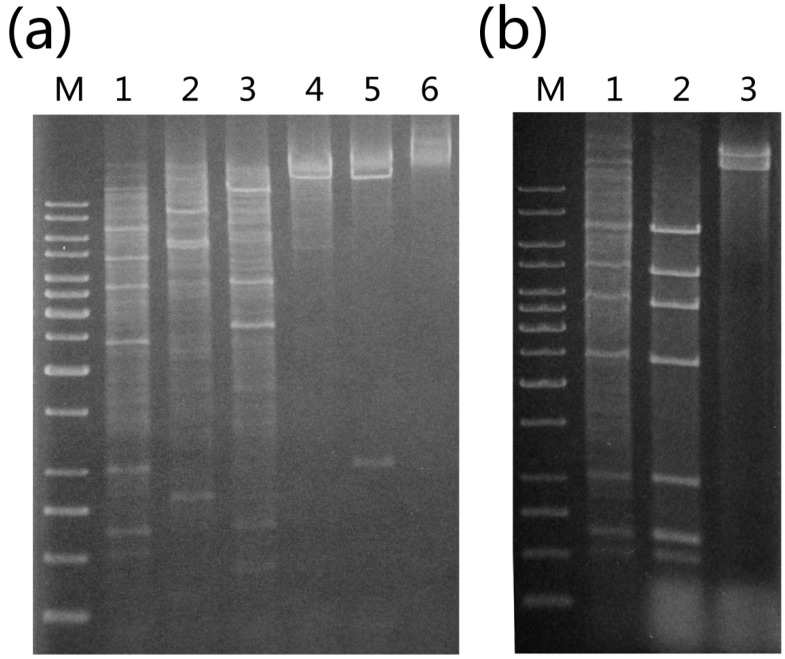
Identification of plasmid pTC1 from *Sulfolobus tengchongensis* RT8-4. (**a**) Restriction digestion of the total DNA from *S. tengchongensis* RT8-4 containing pTC1. The total DNA from the cells was digested with indicated restriction enzymes. Restriction fragments were subjected to electrophoresis in agarose gel. Lane M, 1-kb DNA ladder with sizes of 10, 8, 6, 5, 4, 3.5, 3, 2.5, 2, 1.5, 1, 0.75, 0.5, and 0.25 kb (from top to bottom); lane 1, *Eco*RI; lane 2, *Eco*RV; lane 3, *Pst*I; lane 4, *Xho*I; lane 5 *Sal*I; lane 6, total DNA; (**b**) Restriction digestion of pTC1 DNA. pTC1 DNA was extracted from the cells by alkaline lysis and purified using a plasmid purification kit. The DNA was digested with *Eco*RI. The restriction digest was subjected to electrophoresis in agarose gel. Lane M, 1-kb DNA ladder; lane 1, *Eco*RI digest of the total DNA from *S. tengchongensis* RT8-4 containing pTC1; lane 2, *Eco*RI digest of purified pTC1 DNA; lane 3, purified pTC1 DNA.

We previously reported the isolation and identification of *Sulfolobus tengchongensis* strain RT8-4 from a hot spring in Tengchong in Southwestern China [13]. In a subsequent study, we isolated total DNA from the strain and digested it with various restriction endonucleases. When the digests were subjected to agarose gel electrophoresis and ethidium bromide staining, a stoichiometric pattern of bands with intensity significantly higher than that of smearing bands derived from the genomic DNA of the host was observed, suggesting the presence of an extrachromasomal genetic element in the host cell (Figure 1). This suggestion was supported by the finding that extraction of episomal DNA from *S. tengchongensis* RT8-4 by using the alkaline lysis procedure yielded high molecular weight DNA species that migrated faster than the genomic DNA of the host. To determine whether the DNA was derived from a virus or a plasmid, we examined the culture supernatant of *S. tengchongensis* RT8-4, and no virus-like particles were observed under an electron microscope. Based on these results, we conclude that a plasmid existed in *S. tengchongensis* RT8-4, and designated this plasmid as pTC1.

pTC1 plasmid appeared to exist in high copy number in the original enrichment cultures, but was unstable to plating or sub-culturing. Upon repeated culture transfers, the copy number of pTC1 gradually decreased to levels undetectable even by Southern hybridization. A similar result was obtained for pTC2, the only other pTC plasmid obtained in a colony-purified strain, from a close relative of *S. tengchongenesis* RT8-4 based on 16S rRNA sequence similarity. The conjugational transferability of pTC1 and pTC2 was tested from the appropriate donor strains using the assays described by Schleper *et al.* [1,14] with a plasmid-free *S. tengchongenesis* or *S. solfataricus* strain as a recipient. Despite repeated efforts, however, no conjugation was detected. It is possible that the tested recipient strains were not appropriate for the assays. In addition, pTC plasmids were not integrated into the host genomes, as revealed by Southern hybridization.

### 3.2. Genomic Analysis of pTC1

**Figure 2:**
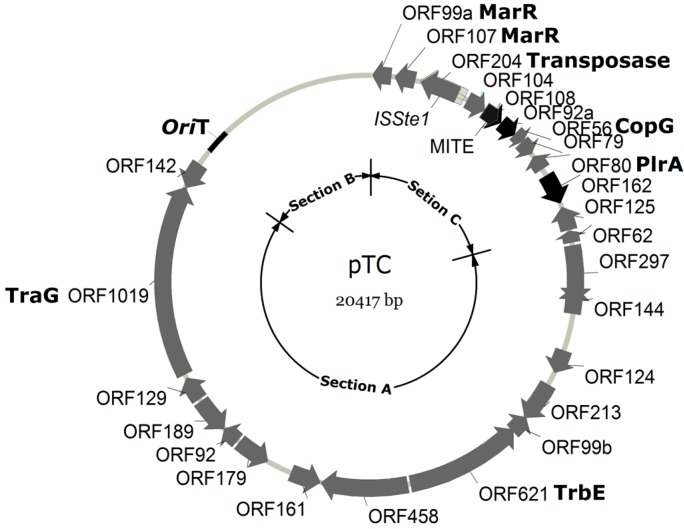
A map of pTC1. ORFs are shown by arrows. ORFs with no homologues in the other known *Sulfolobus* CPs are indicated with blank arrows, whereas those with homologues in other CPs are shown with black arrows. Functional sections A, B and C are indicated.

Both strands of plasmid pTC1 were sequenced. The restriction patterns of the plasmid, predicted from the genomic sequence, agreed well with the results of restriction digestion. As revealed by sequencing, pTC1 is a circular double-stranded DNA molecule of 20,417 bp in length. The G+C content of pTC1 was 41.4%, which was higher than that of its host (34.4%) [13] as well as those of other known *Sulfolobus* CPs. The plasmid contains 26 open reading frames (ORFs) (Figure 2; Table 1). In most of the plasmid, genes are tightly packed or even overlapping. Among all known *Sulfolobus* CPs, pTC1 is the smallest and carries fewest ORFs.

**Table 1:** Genome annotation of pTC1.

Name	Predicted function	Conserved motifs	Homologues in other *Sulfolobus* CPs	Section
ORF62	Transmembrane protein involved in conjugation	Signal peptide and transmembrane regions (1)^1^	pAH1_p35, pARN3_p40, pARN4_p39, pHVE14_p53, pSOG1_p44, pSOG2_p41, pYN01_p2	A
ORF297	Signal peptide involved in conjugation	Signal peptide	pAH1_p1, pARN3_p1, pARN4_p1, pLD8501_p25, pYN01_p50, pHVE14_p3, pING1_p34, pKEF9_p3, pNOB8_p33, pSOG1_p5, pSOG2_p4, pMGB1_p1	A
ORF144	Unknown	-	pAH1_p1, pARN3_p1, pARN4_p1, pLD8501_p25, pYN01_p50, pMGB1_p1	A
ORF124	Unknown	-	pAH1_p1, pARN3_p1, pARN4_p1, pLD8501_p25, pYN01_p50, pMGB1_p1	A
ORF213	The transcription factor TFIIB	TF_Zn_Ribbon superfamily	pAH1_p2, pARN3_p2, pARN4_p2, pLD8501_p24, pYN01_p49, pSOG1_p45	A
ORF99c	Unknown	-	pAH1_p3, pARN3_p3, pARN4_p3, pLD8501_p23, pYN01_p48, pMGB1_p3	A
ORF621	TrbE-like protein involved in conjugation transfer	AAA_10	pAH1_p4, pARN3_p4, pARN4_p4, pHVE14_p1, pING1_p32, pKEF9_p1, pLD8501_p22, pNOB8_p31, pSOG1_p2, pSOG2_p2, pYN01_p47, pMGB1_p4	A
ORF458	Transmembrane protein involved in conjugation	Signal peptide and transmembrane regions (9)	pAH1_p5, pARN3_p5, pARN4_p5, pLD8501_p6, pSOG1_p7, pYN01_p36, pMGB1_p5	A
ORF161	Transmembrane protein involved in conjugation	Transmembrane regions (2)	pAH1_p6, pARN3_p6, pARN4_p6, pYN01_p35	A
ORF179	Transmembrane protein involved in conjugation	Transmembrane regions (3)	pAH1_p9, pARN3_p7, pARN4_p8, pLD8501_p1, pYN01_p33	A
ORF92	Transmembrane protein involved in conjugation	Transmembrane regions (2)	pAH1_p10, pARN3_p8, pARN4_p9, pYN01, pMGB1_p8	A
ORF189	Transmembrane protein involved in conjugation	Signal peptide and transmembrane regions (1)	pAH1_p11, pARN3_p9, pARN4_p10, pSOG1_p4, pYN01_p31, pMGB1_p9	A
ORF129	Transmembrane protein involved in conjugation	Signal peptide and transmembrane regions (2)	pAH1_p12, pARN3_p10, pARN4_p11, pYN01_p30, pMGB1_p10	A
ORF1019	VirD/TraG-like protein	TraG_VirD4/AAA_10	pAH1_p13, pARN3_p11, pARN4_p12, pHVE14_p14, pING1_p5, pKEF9_p12, pNOB8_p10, pSOG1_p12, pSOG1_p2, pSOG2_p12, pYN01_p29, pMGB1_p11	A
ORF142	Unknown	-	pAH1_p14, pARN3_p12, pARN4_p13, pING1_p6, pKEF9_p13, pSOG1_p13, pSOG2_p13	A
ORF99a	Predicted transcriptional regulators	HTH_ARSR superfamily	pING_p18, pNOB8_p18, pNOB8_p44, pYN01_p41, pMGB1_p20	C
ORF107	Predicted transcriptional regulators	HTH_MARR	pAH1_p22, pKEF9_p24, pNOB8_p18, pNOB8_p2, pNOB8_p19, pYN01_p42, pLD8501_p16, pMGB1_p20, pMGB1_21	C
ORF204	Transposase	COG3316	pARN4_p16	C
ORF104	Unknown	-	pARN3_p27, pARN4_p25, pING1_p19, pNOB8_p20, pYN01_p17, pHVE14_p30, pKEF9_p25, pSOG1_p23, pSOG2_p26	C
ORF108	Unknown	-	-	C
ORF92a	Unknown	-	-	C
ORF79	Unknown	-	pSOG1_p24, pSOG2_p27, pMGB1_p7	C
ORF80	PlrA-like protein	*Sulfolobus*_pRN	pING1_p26, pARN4_p33, pNOB8_p28, pKEF9_p33, pSOG1_p34, pLD8501_p29, pHVE14_p41, pAH1_p33, pYN01_p6	C
ORF56	CopG family transcriptional regulator	PHA01748/RHH_1 superfamily	pAH1_p28, pKEF9_p28, pYN01_p11, pNOB8_24, pMGB1_p26	C
ORF162	Unknown	-		C
ORF125	Unknown	-	pARN3_p38, pARN4_p37, pKEF9_p38, pHVE14_p50, pSOG1_p42, pSOG2_p39, pYN01_p4	C

^1^ The number of transmembrane regions in a protein encoded by an ORF is indicated by the number in parentheses.

To determine the similarity of pTC1 ORFs to homologous ORFs from other CPs, we assembled a database consisting of ORFs from 12 other known *Sulfolobus* CPs (*i.e.*, pNOB8, pING1, pARN3, pARN4, pAH1, pSOG1, pSOG2, pLD8501, pYN01, pKEF9, pHVE14 and pMGB1). As revealed by BLASTP with an E-value cutoff of <10^−3^, only three ORFs from pTC1 (*i.e.*, ORF108, ORF92a and ORF162) have no homologues in the other twelve *Sulfolobus* CPs. All the three ORFs are located in section C of the plasmid genome (see below). The rest of the pTC1 ORFs share 22.50%~69.99% similarity at the amino acid sequence level with their respective best matches in the other 12 CPs.

Genomic comparison shows that pTC1 resembles known *Sulfolobus* CPs in architecture [6,9]. Like all other *Sulfolobus* CPs, pTC1 comprises three conserved and functionally distinct genomic sections (Figure 2 and Table 1). Section A of pTC1 accounts for about half of the plasmid (~12.5 kb) and contains 15 ORFs that are conserved among *Sulfolobus* CPs. All section A proteins, except for those encoded by ORF144, 124, 213, 142 and 99b, are predicted to possess transmembrane helix motifs, and are believed to be involved in plasmid conjugation. These include two highly conserved *Sulfolobus* CP proteins that are likely homologues of the bacterial conjugative ATPase proteins VirD/TraG (ORF621) and TrbE (ORF1019). Bacterial TraG is the coupling protein that connects the membrane-spanning mating pair formation (Mpf) complex with the cytoplasmic nucleoprotein relaxosome complex at the cytoplasmic membrane during conjugative transfer of DNA [15]. However, it is worth noting that no genes encoding relaxase, a bacterial homologue which is involved in the mobilization of the conjugational plasmid transfer [9], were found in the pTC1 genome. Also unusual was the observation that ORFs probably involved in transcriptional regulation (*i.e.*, ORF144, 124, 213 and 99b) interrupted the otherwise contiguous cluster of ORFs encoding transmembrane proteins. The contiguous array of these proteins is highly conserved among other *Sulfolobus* CPs.

**Figure 3:**
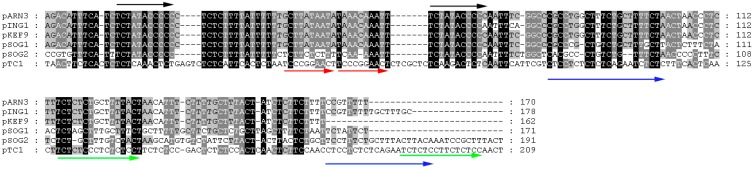
Alignment of the putative origins of replication of pARN3, pING1, pKEF9, pSOG1, pSOG2 and pTC1. Repeating sequences are indicated by arrows. Identical or similar sequences are shown in dark or grey background, respectively.

Section B contains multiple sequence repeats and no ORFs. Based on Z-curve and GC-skew analyses, the origin of replication for pTC1 is located in a 209-bp stretch, downstream of ORF142, in a large intergenic region (Figure 2). The 209-bp region shares limited sequence similarity to the replication origin of pSOG2, pKEF9, pING1, pARN3, and pSOG1 (Figure 3). However, the conserved sequence TCTATACCCCC, found in the putative origin of the other CPs [9], is not present in pTC1 (Figure 3). On the other hand, there are two perfect direct repeats (TCCCCGGAACT) and three imperfect direct repeats (TCTCTCCNNCT) in this region as well as four imperfect repeats (AGCAGCGCTTGYCCT) 529–959 bp downstream of this region. The presence of these direct and inverted repeating sequences supports the possibility that pTC1 initiates replication in this region [16].

Section C, which includes a tightly packed cluster of six to nine genes, is conserved among previously characterized *Sulfolobus* CPs and has been implicated in the initiation and regulation of plasmid DNA replication [7,9]. These genes encode hypothetical relaxase (e.g., some pING ORFs), putative RepA (pING1_ORF99, pKEF9_ORF106), …, various types of transcriptional regulators, such as CopG (pAH1_p28, pKEF9_p28, pYN01_p11, pNOB8_24, pMGB1_p26), MarR-like protein(pAH1_p22, pKEF9_p24, *etc.*), Zinc finger protein (pAH1_p36), *etc.*, as well as 1–4 hypothetical proteins. Surprisingly, section C in pTC1 is not well conserved as compared to the corresponding region in other *Sulfolobus* CPs. Most of the CPs encode a putative RepA protein. However, no homologue of a gene encoding putative RepA was found in pTC1. A *plrA* gene was identified in pTC1. PlrA, a highly conserved transcriptional regulator in *Sulfolobus* plasmids, is speculated to be involved in plasmid segregation [9]. All previously described CPs contain an ORF for a pNOB8-type integrase of the tyrosine recombinase superfamily. However, no homologue of the integrase gene was found in pTC1. Instead, an insertion element and a miniature inverted-repeat transposable element (MITE) were detected in section C (see below).

**Figure 4:**
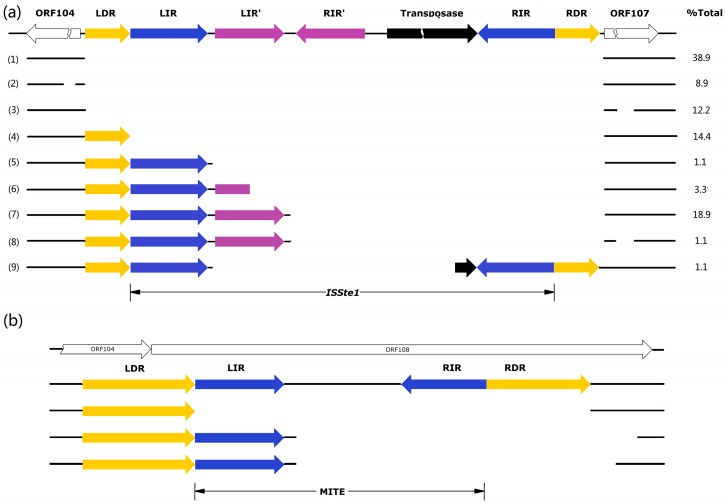
Patterns of deletion at the sites of transposition by *ISSte1* (**a**) and MITE (**b**). Total DNAs or plasmid DNAs were isolated from enrichment cultures established with various hot spring and mud hole samples. PCR targeting the *ISSte1* site or the MITE site was carried out. Fragments shorter than expected for the insertion of full-length *ISStel* or MITE into the pTC1 DNA were sequenced. Repeating sequences are labeled in color and specified. ORFs are shown with blank arrows. An additional deletion (AGGGGCTCT) occurred at 88 bp upstream of LDR in type 2 deletion variants, whereas an additional deletion (AGAGACCGAGAATGATA) was 86 bp downstream of RDR in types 3 and 8 deletion variants. The percentage of each deletion type for *ISStel* in all samples is indicated.

The insertion sequence (IS), denoted *ISSte1* [17], is 762 bp in length and located in the intergenic region between ORF107 and ORF104 (Figure 4a). It contains a transposase (Tpase) gene (ORF204) flanked by 16-bp perfect inverted repeats (IR: GTGTGTGTCCAACAAT). Notably, there is a pair of perfect inverted repeats (IR': GGGTCAGGACGGG) between the left IR and ORF204. The insertion of *ISSte1* has led to the duplication of the target site of 8 bp (DR: ATCACAAA). Sequence analysis shows that ORF204-encoded Tpase contains the conserved DDE domain (Figure S1) [17]. This, together with the size and sequence of the IRs, places *ISSte1* in the *IS6* family [17].

MITEs are mobile genetic elements derived from ISs with DDE Tpases, and contain flanking terminal IRs but no internal Tpase gene [17,18,19]. The MITE sequence identified in pTC1 is only 142 bp long (Figure 4b). Intriguingly, the entire element resides in ORF108. It would be of interest to determine if ORF108 is functional. If the ORF encodes a functional protein, the presence or absence of the MITE at the site of transposition might be of significance to the biology of the plasmid. It is clear, however, that ORF108 is not essential for the plasmid since it is absent from all other known *Sulfolobus* CPs. The MITE of pTC1 carries long imperfect IRs (LIR: AATCGAGAACGGTGTCAT, RIR: ATGAGACATTCCTCGTT) at the two ends and is bounded by long imperfect DRs (LDR: AAGTGAGGTGATGTAAATGGTCAC, RDR: CAGTATATCATGTACTGGTAC. The intervening sequence between the two flanking IRs shares no significant similarity with known sequences in the public database, indicating that the element is a type II MITE. Since both IRs and DRs of the MITE are not well conserved, it appears that the element has lost the mobility due to mutation.

### 3.3. pTC Plasmids in the Geothermal Area of Tengchong

**Figure 5:**
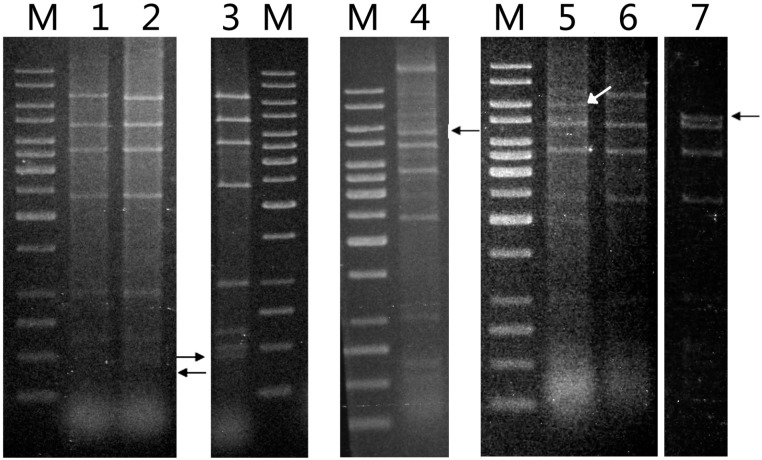
Restriction patterns of pTC plasmids. Plasmid DNAs were extracted from various isolates by alkaline lysis and purified using a plasmid purification kit. The DNAs were digested with *Eco*RI. The restriction digests were subjected to electrophoresis in agarose gel. Lane M, 1-kb DNA ladder with sizes of 10, 8, 6, 5, 4, 3.5, 3, 2.5, 2, 1.5, 1, 0.75, 0.5, and 0.25 kb (from top to bottom); lane 1, pTC1 DNA from *S. tengchongensis* RT8-4; lane 2, plasmid DNA from isolate H7; lane 3, plasmid DNA from isolate H7; lane 4, plasmid DNA from isolate H3; lane 5, plasmid DNA from isolate D2; lane 6, plasmid DNA from isolate D4; lane 7, plasmid DNA from isolate H5. Restriction fragments containing a deletion are indicated by arrows.

To determine the distribution of pTC1 and its variants in acidic hot springs and mud holes in Tengchong, we collected 35 samples at various spots in a general area of ~0.1 km^2^. Enrichment cultures from 27 of the samples were successfully established in Zillig’s medium supplemented with 0.2% tryptone [3]. Plasmid DNAs were isolated from 11 of the enrichment cultures by using the alkaline lysis method. Restriction analysis showed that the *Eco*RI cleavage patterns of seven of the plasmids resembled that of pTC1, whereas those of the other four were also indistinguishable from that of pTC1 except for one fragment (Figure 5). An additional fragment of ~400 bp appeared in one of the pTC variants, whereas the 6.8-kb *Eco*RI fragment, which harbored *ISStel*, was shortened to 6.0, 5.5 and 5.2 kb, respectively, in the other three variants. The ~400-bp *Eco*RI fragment of the former pTC variant, denoted pTC2, was sequenced. As shown in Figure 4 and Figure S2b in supplementary, pTC2 was 163 bp shorter than pTC1 in that fragment, and the MITE found in pTC1 was absent from pTC2 except for a single DR of the element. Therefore, pTC1 appears to have resulted from the insertion of the MITE in pTC2. These results suggest that pTC plasmids were widely distributed in the geothermal area of Tengchong, and pTC1 was likely a predominant species of these plasmids.

The presence of the insertion sequences in pTC1 prompted us to look for naturally-occurring variants of this plasmid. PCR reactions were carried out on total DNA or plasmid DNA samples isolated from each enrichment culture using a pair of primers targeting sequences upstream and downstream of *ISStel*. The primers were designed based on the sequences inside the upstream ORF104 and downstream ORF107 to ensure specific amplification of the sequence from pTC plasmids. Because of the absence of the integration of pTC into the host genome, it would be most likely that the specific PCR products were derived from the sequences of free pTC plasmids. A total of 90 PCR fragments shorter than that expected for the transposition by a full-length *ISStel* were obtained and subsequently sequenced. These sequences fall into nine categories (Figure 4 and Figure S2a in supplementary). Only a small fraction (14.4%) of the fragments contained the unaltered plasmid sequence, suggesting that either no transposition by *ISStel* had occurred at the site or an inserted *ISStel* had undergone precise excision, leaving behind a single copy of the target sequence (*i.e.*, DR). In any case, the target site appears to be efficiently used for *ISStel* transposition. No perfect reversion of the transposition by *ISStel* to the target site was observed. In the remainder of the fragments, transposition of *ISStel* to the target site and subsequent excision of the inserted IS in an imprecise manner were detected. A large deletion spanning the entire *ISStel* as well as both DRs and a 14-bp 3'-flanking sequence was found in 70.1% of these fragments. Some of them carried a small additional deletion either upstream (17.4%) or downstream (12.7%) of the target site for *ISStel* transposition. The rest of these fragments (29.8%) had smaller deletions, which started 8–65 bp downstream of LIR and mostly ended 5–19 bp downstream of RDR. So all of them retained LDR and LIR, but lost the Tpase gene. Taken together, these observations suggest that deletion of *ISStel* proceeded more frequently in an imprecise manner than in a precise manner in natural environments.

A diverse array of pTC variants associated with the transposition of MITE and subsequent deletion of the inserted element was also observed. As shown in Figure 4 and Figure S2b in supplementary, the MITE sequence was either entirely absent or deleted in different fashions. The sequence lacking the entire MITE may have yet to undergo transposition by the MITE. It is also possible that the sequence resulted from the precise deletion of the inserted MITE form a pTC1-like plasmid. Since the LIR of the pTC1 MITE is identical to the target sequence, the divergence between the two inverted repeats suggests that the MITE in pTC1, acquired through transposition, has probably become immobilized through mutation of the RIR. Imprecise deletion of the MITE apparently occurred as observed with *ISStel*. 

## 4. Discussion

A large number of extrachromosomal genetic elements have been isolated from members of the family Sulfolobaceae thriving in various geothermal areas around the globe. In our previous study, we showed that *S. tengchongensis* RT8-4, isolated from a hot spring in Tengchong, China, was infected by the spindle-like virus STSV1 [20]. Subsequent analyses led to the identification of the plasmid pTC1 in the same *Sulfolobus* isolate. In our surveys, pTC1 and its variants were identified from 11 out of 27 enrichment cultures established with samples collected from various spots in the geothermal area of Tengchong. When a more sensitive PCR-based assay targeting the pTC1 sequences flanking *ISStel* was employed, 14 out of 20 samples produced positive results. Therefore, pTC plasmids are widespread in the Tengchong area. No other plasmids were found in our surveys.

The apparent conservation of pTC1 section A, especially the presence of ORFs encoding bacterial homologues of VirD/TraG (ORF621) and TrbE (ORF1019), suggests that pTC1 and its variants are conjugative plasmids. However, despite repeated efforts, we were unable to demonstrate the conjugational transfer of the plasmids under our experimental conditions. The lack of conjugation activity might be attributed to the absence of an identifiable relaxase-encoding gene in pTC plasmids. However, this argument is weakened by the finding that several known *Sulfolobus* CPs lacking the relaxase (e.g., pSOG1, 2) were capable of conjugational transfer [21,22]. It is possible that pTC plasmids lack other ORFs or *cis* elements required for conjugational transfer. It is also possible that the conjugation assay conditions, e.g., selection of recipient strains, were inadequate for the detection of the plasmid transfer. Therefore, more tests are needed before a conclusion can be drawn.

The copy number of pTC1 in the cells in the original enrichment cultures was high but rapidly decreased upon sub-culturing. pTC1 differs most significantly from other known *Sulfolobus* CPs in section C of the plasmid, which is proposed to encode proteins for plasmid DNA replication. For example, unlike many other known *Sulfolobus* CPs, pTC1 possesses no *repA* gene. The absence of a RepA homologue in pTC1 suggests that this protein is probably not essential for the replication of the plasmid. This and the observation that RepA was also missing from pSOG plasmids [9] suggest that an unidentified protein may serve as a replication initiator in *Sulfolobus* CPs lacking RepA. In addition, pTC1 encodes no Par-like proteins, which are involved in partitioning of bacterial plasmids. This provides a possible explanation for the instability of pTC plasmids during repeated culture transfers.

Mobile elements (*i.e.*, *ISSte1* and MITE) play an important role in generating the diversity of pTC plasmids. *ISSte1* appears to be efficiently inserted into pTC plasmids in nature since no plasmids that had not undergone the transposition were isolated from over 30 hot spring and mud hole samples. Interestingly, the inserted *ISSte1* was subjected to inactivation and removal in a range of manners, leaving behind various forms of IS relics. However, in no relics was a Tpase-encoding gene found. Perfect reversal of the transposition process, which would have left two DRs in tandem in the resulting plasmid, was not detected. The insertion element in only a small fraction (14.4%) of the plasmids containing various forms of IS relics was removed precisely, returning the insertion site to the pre-transposition state. As observed for the spontaneous deletion in bacteria, the non-tandem DRs at the boundaries of the inserted *ISSte1* probably promoted the precise excision by host-encoded functions [23,24]. The low frequency of the event is not unexpected in view of the finding that precise excision of the ISs was rarely detected in *Sulfolobus* [25]. The remaining relics presumably resulted from imprecise excision of inserted *ISSte1* in different manners. Degradation of *ISSte1* in an imprecise mode did not seem to take place randomly but preferentially yielded relics retaining 5'-end parts of the *ISSte1*. The molecular mechanisms for the processes are unclear, but they may include abortive transposition by transposase [26]. It is equally possible that unknown host defense systems are involved in the removal of *ISSte1*. The MITE in pTC1 apparently underwent removal in a similar fashion. Taken together, our results are consistent with the notion [27] that the host cell may have developed a strategy to maintain a balance between the insertion and elimination of mobile genetic elements to permit genomic plasticity while inhibiting their fast spreading.

## Supplementary Materials

Supplementary materials can be accessed at: http://www.mdpi.com/2075-1729/5/1/506/s1.
