# Supplementary material for: pTC Plasmids from Sulfolobus Species in the Geothermal Area of Tengchong, China: Genomic Conservation and Naturally-Occurring Variations as a Result of Transposition by Mobile Genetic Elements
Source: Life (Basel). 2015 Feb 12;5(1):506–20. doi: 10.3390/life5010506 (PMC4390865; doi:10.3390/life5010506)
Supplement: Supplementary file 1 [file life-05-00506-s001.pdf]

Supplementary Materials

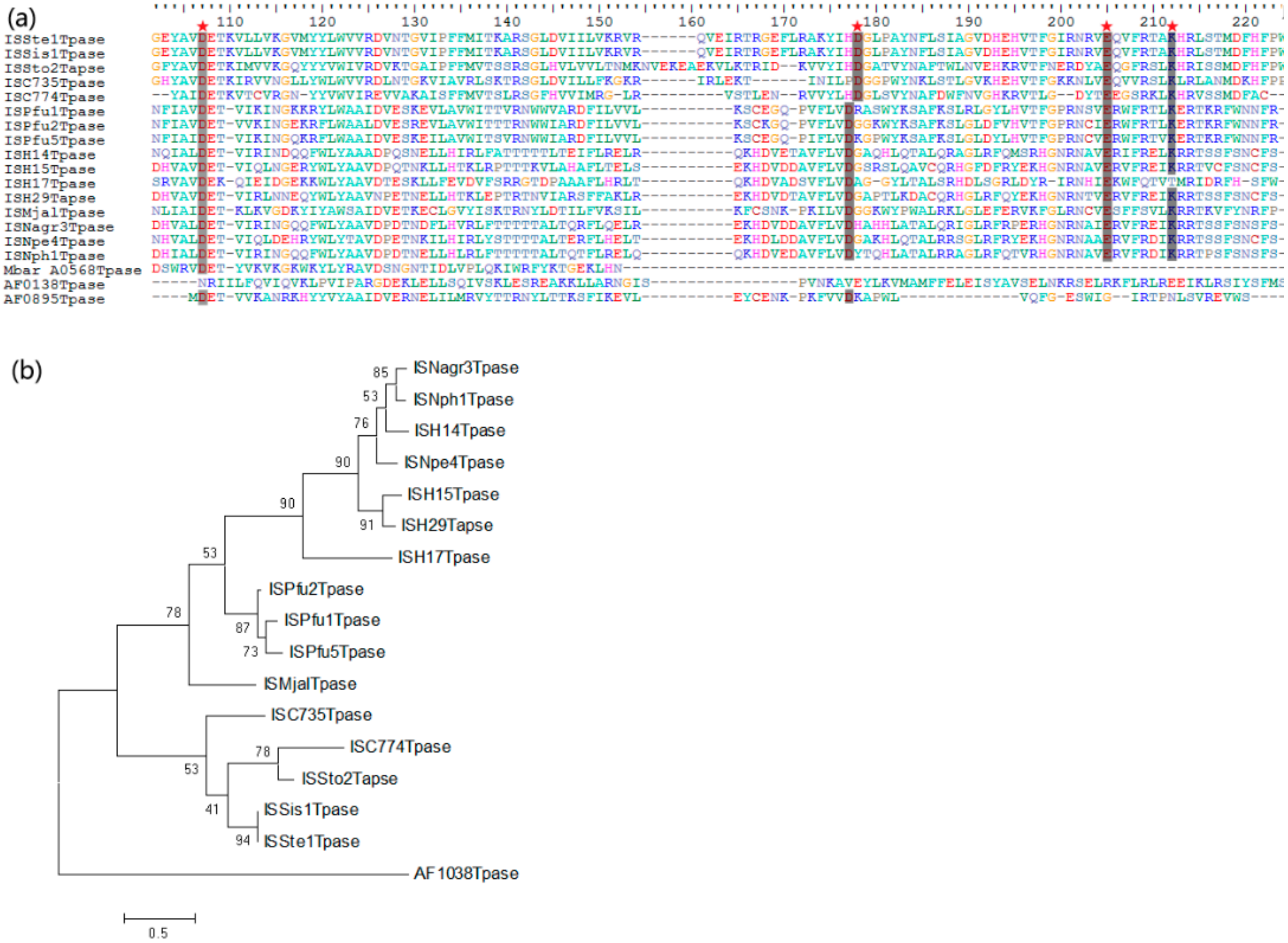

Figure S1. (**a**) Sequence alignment of the conserved DDE domains of transposases of the *IS6* family. Amino acid residues D…D…E…K are shown in grey background (**b**) Phylogenetic tree of transposases of the *IS6* family.

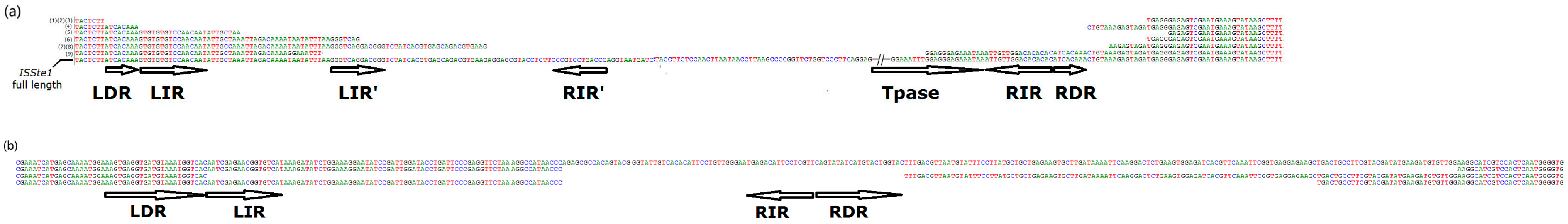

Figure S2. Sequences of deletions at the sites of transposition by *ISSte1* (**a**) and MITE (**b**). See Figure 5 for deletion categories.
